# The Lords' Committee

**Published:** 1891-02-21

**Authors:** 


### THE LORDS' COMMITTEE.

On February 12th the arrangements at the Middlesex Hospital were under examination, and, as Lord Sandhurst is president of this hospital, he left the duty of questioning chiefly to Lord Kimberley.

Mr. F. C. Melhado, Secretary to the Middlesex Hospital, said that he had held that post for three years, having previously been assistant secretary for nine years. The hospital was governed by the Board of Governors and a weekly committee appointed annually by the Governors, which consisted of 24 members. None of the medical officers were on the Board of Governors, who held quarterly meetings, as they were ineligible for election. The hospital contained 307 beds, of which 24 were set apart specially for cancer, there being an endowment for that particular purpose; and of the general hospital beds, 250 to 260 were on an average occupied daily. Witness had the general control of the establishment and could suspend or dismiss servants, but the nurses and female staff were under the control of the lady superintendent, who had similar powers of dismissal and suspension, subject to the approval of the weekly committee. Replying to the Chairman, witness gave the annual cost of each occupied bed as £87 12s. 1½d. Asked why it should be more than St. George's, which was £75, he said it depended upon the basis on which the calculation was made. Nothing was refused to the patients. They came in and stayed until they recovered or died. He thought it might be advantageous to have a board of control for all hospitals.

Mr. Edward Fardon, resident medical officer at the hospital, was next called. He stated that his salary was £200 a-year, with board and lodging, and he had held the office for 12 years. He had a general medical supervision of the hospital, so far as sanitary arrangements, ventilation, and warming were concerned. He was responsible for admissions, and also for the medical care of the nurses and servants, and he exercised a general authority over the wards. There were within a radius of a mile eight general hospitals and 26 special hospitals. These had altogether 2,050 beds, including the 307 in the Middlesex Hospital. The Chairman: With all this enormous amount of accommodation for the sick, do you ever have to send people away for want of room? Oh, yes; if we had room we could take in many more. Is your out-patient department ever crowded? Inconveniently so. There was no restriction on the admission whatever. The hospital was practically free to all necessitous people who liked to apply within certain hours.

Mr. Alfred Pearce Gould, of the Medical School of the Middlesex Hospital, was next called. He stated that the number of students varied from 250 to 300. They usually had about 65 occasional students, who stayed for three or six months. The fee for a full medical curriculum of four years was 100 guineas on entrance and 110 guineas paid in instalments. During the last few years the fees had amounted to about £5,000, and the expenses had been £2,000, leaving £3,000 to be divided amongst the medical teachers.

On Monday, Lord Sandhurst in the chair, Dr. Thorne, of the Local Government Board, came forward to give his views of the sanitary state of St. Bartholomew's; his report concluded: "First, the sanitary circumstances of the Nurses' Home in Little Britain, are such as to forbid the belief that they can in any way have been concerned in the production of the diseases; secondly, that while I am unable to speak with equal confidence as to the Duke Street Home, I have no grounds for believing that diphtheria has been induced by any of the sanitary conditions of that building. That as regards the drainage of the ward blocks, and the internal arrangements of these buildings, conditions do exist, and it is notable in the ward kitchens, which do tend to unwholesomeness, and which have been known to produce sore throat, and that even if this form of sore throat be not in its beginning regarded as of a specific character, it is certain that those suffering from it are to an exceptional degree liable to contract diphtheria when the disease is prevalent. This statement is not intended to prejudge the question as to the origin and diffusion of infection during the recent prevalence, as to which the necessary data are not forthcoming." Mr. Cross and Mr. I'Anson also spoke on the subject of drainage. Mr. Robert Barnes, F.R.C.P. and F.R.C.S., called next, stated that he was connected with a number of hospitals, both general and special. He considered special hospitals absolutely necessary to supplement the work of the general hospitals. There were six supplementary general hospitals without medical schools, namely, the French, Italian, German, Great Northern, Central, Metropolitan, and the West London Hospitals. Besides these there were in London ten hospitals for children, containing 505 beds; one for children suffering from hip disease, with 50 beds; four for diseases of women, with 225 beds; four for consumption, with 615 beds; the London Fever Hospital, 200 beds; and five metropolitan fever hospitals, under public management, with 2,141 beds; a small-pox hospital, 108 beds; one for heart diseases, 26 beds; three for incurables, 301 beds; Hospital of St. John and Elizabeth, 50 beds; the London Lock Hospital, 240 beds; three for lunatics, with 656 beds; six lying-in hospitals, 175 beds; five ophthalmic hospitals, 195 beds; three orthopædic hospitals, 118 beds; one for stone, with 24 beds; the Seamen's Hospital, 225 beds, and a branch in the Albert Dock, with 20 beds; Poplar Hospital for Accidents, 51 beds; four for diseases of the throat and ear, with 52 beds; three for skin diseases, with 52 beds; one Cancer Hospital, with 120 beds; and two for fistula, with 42 beds. That list gave a total of 6,494 beds, but he did not wish to say it was a complete list. He considered that special hospitals, besides affording better treatment to the patients, afforded medical men a better opportunity to study special diseases than was afforded at general hospitals. The witness said he was decidedly against any State control of charities, nor would he at present like to trust the County Council.

